# Identification of 6 dermatomyositis subgroups using principal component analysis‐based cluster analysis

**DOI:** 10.1111/1756-185X.13609

**Published:** 2019-06-09

**Authors:** Huiyi Zhu, Chanyuan Wu, Nan Jiang, Yanhong Wang, Jiuliang Zhao, Dong Xu, Qian Wang, Mengtao Li, Xiaofeng Zeng

**Affiliations:** ^1^ Department of Rheumatology, Peking Union Medical College Hospital, Peking Union Medical College and Chinese Academy of Medical Sciences and Key Laboratory of Rheumatology and Clinical Immunology Ministry of Education Beijing China; ^2^ Department of Epidemiology and Biostatistics, Institute of Basic Medical Sciences, Chinese Academy of Medical Sciences School of Basic Medicine Peking Union Medical College Beijing China

**Keywords:** cluster analysis, dermatomyositis, principal component analysis, subgroups

## Abstract

**Objective:**

Dermatomyositis (DM) is a heterogeneous disease with a wide range of clinical manifestations. The aim of the present study was to identify the clinical subtypes of DM by applying cluster analysis.

**Methods:**

We retrospectively reviewed the medical records of 720 DM patients and selected 21 variables for analysis, including clinical characteristics, laboratory findings, and comorbidities. Principal component analysis (PCA) was first conducted to transform the 21 variables into independent principal components. Patient classification was then performed using cluster analysis based on the PCA‐transformed data. The relationships among the clinical variables were also assessed.

**Results:**

We transformed the 21 clinical variables into nine independent principal components by PCA and identified six distinct subgroups. Cluster A was composed of two sub‐clusters of patients with classical DM and classical DM with minimal organ involvement. Cluster B patients were older and had malignancies. Cluster C was characterized by interstitial lung disease (ILD), skin ulcers, and minimal muscle involvement. Cluster D included patients with prominent lung, muscle, and skin involvement. Cluster E contained DM patients with other connective tissue diseases. Cluster F included all patients with myocarditis and prominent myositis and ILD. We found significant differences in treatment across the six clusters, with clusters E, C and D being more likely to receive aggressive immunosuppressive therapy.

**Conclusion:**

We applied cluster analysis to a large group of DM patients and identified 6 clinical subgroups, underscoring the need for better phenotypic characterization to help develop individualized treatments and improve prognosis.

## 1 INTRODUCTION

Dermatomyositis (DM) is an idiopathic inflammatory myopathy (IIM) characterized by inflammatory disorders primarily affecting the skeletal muscle and skin with typical cutaneous lesions.^1^ The outcomes of IIM are poor, with a 5‐year survival rate of less than 50%.^2^ The diagnosis of DM is still based on the Bohan and Peter criteria,^3^, ^4^ which were proposed in 1975. Four of these five criteria are related to muscle involvement, and the fifth is the presence of typical cutaneous lesions. In recent years, DM has been shown to be a heterogeneous disease entity with a wide range of clinical features. In addition to muscle and skin involvement, other organs are often involved, leading to arthritis, esophageal disease, interstitial lung disease (ILD), and cardiac damage.^5^ Patients with DM also have a higher risk of malignancy than the general population, and 10%–40% of DM patients go on to develop a malignancy.^1^, ^6^ Furthermore, myositis‐specific antibodies have been associated with certain clinical features of DM, the malignancy risk in DM, and the response of DM patients to treatment,^7^ and hence, may help with the diagnosing and subtyping of DM.

The identification of clinical DM phenotypes has been reported on extensively in recent years. Bohan and Peter^3^, ^4^ suggested four subtypes of DM: idiopathic DM, juvenile DM, DM associated with cancer, and DM associated with other connective tissue diseases. Recent studies have identified a new DM subtype: clinically amyopathic DM (CADM), which includes amyopathic DM, wherein the disease affects only the skin, and hypomyopathic DM, wherein cutaneous manifestations are associated with evidence of subclinical myositis.^1^ In the past, classifications of DM were mostly developed based on researchers’ clinical experiences and lacked solid data support. In addition, older classification systems did not take into account the involvement of other vital organs, as in ILD and cardiac involvement, which reduces the prognostic and therapeutic values of these classifications.

Different DM subtypes have distinct clinical manifestations, responses to therapy and prognoses. For example, muscle disease is much more responsive to systemic corticosteroids than the skin component.^8^ Patients with ILD may respond well to cyclophosphamide or mycophenolate mofetil.^9^ Survival has been reported to be the worst (25% at 5 years) in cancer‐associated myositis, followed by CADM (61% at 5 years).^10^ Moreover, ILD with mildly increased serum creatine kinase (CK) levels and skin ulcers are independent risk factors for death in DM patients.^10^ Tailoring the therapeutic strategy according to the DM subtype may improve the survival of DM patients. Precise phenotyping is critical for the development of individualized treatments and also for understanding the underlying pathological mechanisms.

In the present study, we aimed to objectively identify the subtypes of DM by using a new exploratory statistical method. We applied principal component analysis (PCA)‐based cluster analysis to identify DM subtypes based on characteristic clinical manifestations and to determine the relationships between these variables. This methodology identified six distinct DM subtypes with different clinical characteristics. The validity of the clustering was confirmed by the significant differences in immunosuppressive therapies across the six subgroups.

## 2 METHODS

### 2.1 Study population

This study retrospectively enrolled 794 patients diagnosed with DM or CADM from both the out‐patient clinic and in‐patient wards of Peking Union Medical College Hospital between January 2012 and August 2016. DM was diagnosed according to the Bohan and Peter criteria for probable or definite DM,^3^, ^4^ while CADM was diagnosed according to the Sontheimer criteria.^11^ Patients with polymyositis (PM) were excluded because PM and DM are well recognized as two distinct subtypes, and because the PM patient population is a potentially mixed group with uncertain diagnoses due to low rates of muscle biopsy, especially among patients treated in out‐patient clinics. Other myopathies with an identifiable etiology were also excluded. The need for ethics approval and informed patient consent was waived because the study involved a retrospective review of patient records.

### 2.2 Data extraction

From the patients' medical charts, we extracted the data collected at the time of the first hospitalization or the first clinic visit in our hospital after the confirmation of the diagnosis. For all patients, we retrospectively reviewed data on the following parameters: demographics, IIM‐related clinical manifestations and laboratory findings, cumulative major organ involvement, and immunosuppressive therapy. Malignancy was documented if it occurred within 3 years before or after the diagnosis of DM. ILD was determined using high‐resolution computed tomography.^12^ Cardiac involvement, including systolic or diastolic dysfunction, pericarditis, and pericardial effusion, was evaluated using echocardiography and electrocardiography. We also documented the administration of aggressive immunosuppressive therapy, which was defined as a daily glucocorticoid dose equivalent to or more than 0.5 mg/kg prednisone, and treatment with cyclophosphamide, mycophenolate mofetil, cyclosporine, or tacrolimus.

### 2.3 Data analysis protocol

Cluster analysis, the most popular method of unsupervised learning, is a multivariate technique used for identifying subgroups sharing similar characteristics in a data set.^13^ In this study, cluster analysis was performed to identify subgroups among DM patients. We followed four critical steps in performing the statistical analysis: selection of clinical variables for analysis, cluster analysis of these variables to explore the relationships between them, PCA to reduce interactions between the variables, and cluster analysis of patients based on the PCA‐transformed data.

Categorical variables are presented as numbers (percentages), and continuous variables are presented as mean (standard deviation) or median (interquartile range) depending on whether their distribution was normal or skewed. All analyses were conducted using SPSS version 24.0 for Mac (IBM).

### 2.4 Variable selection

In our study, variables with the same clinical significance, such as the V‐sign and shawl sign, and myalgia and muscle tenderness, were combined into new variables for analysis. Variables with a large number of missing data, such as elevated gamma glutamyl transpeptidase (GGT), alkaline phosphatase (ALP), and lactate dehydrogenase (LDH), were excluded from further analysis. In total, 21 variables were included in the analysis (Table 1). Continuous variables, such as age at onset and CK level, were standardized. Seventy‐four patients with missing data for these 21 variables were excluded, which is necessary for PCA and cluster analysis. This resulted in an analytic population of 720 patients (91% of the initial study population). We compared the characteristics of the patients who were included in our study with those of the patients who were excluded from our study (Table S1), and found that most of the clinical features studied did not differ between these two groups.

**Table 1:** Clinical characteristics of 720 patients with dermatomyositis

	Patients, n (%) (N = 720)
Demographics	
Female^a^	522 (72.5)
Age at onset,^b^ years	46.3 (14.6)
Course of disease,^a^, ^c^ months (n = 694)	9.0 (30.0)
Clinical features	
Muscle weakness	560 (77.8)
Myalgia/muscle tenderness	409 (56.8)
Heliotrope rash	512 (71.1)
Gottron sign	329 (45.7)
V‐sign/shawl sign	405 (56.3)
Mechanic's hand	72 (10.0)
Raynaud phenomenon	78 (10.8)
Periungual telangiectasia	36 (5.0)
Digital ulcer	26 (3.6)
Calcinosis cutis	4 (0.6)
Fever	191 (26.5)
Arthritis/arthralgia	246 (34.2)
Interstitial lung disease	383 (53.2)
Respiratory symptoms as an initial manifestation	82 (11.4)
Pericarditis/pericardial effusion	45 (6.3)
Myocarditis	10 (1.4)
Esophageal involvement	131 (18.2)
Comorbidities	
Malignancy	31 (4.3)
Other connective tissue disease	39 (5.4)
Laboratory data	
Creatine kinase level,^c^ U/L	161.5 (697.5)
Elevated GGT or ALP^d^ (n = 643)	249 (38.7)
Elevated LDH^d^ (n = 659)	537 (77.3)
Usage of aggressive immunosuppressive therapy^a^	238 (33.1)

Abbreviations: ALP, alkaline phosphatase; GGT, gamma glutamyl transpeptidase; LDH, serum lactate dehydrogenase.

a Variables not used for the creation of clusters.

b Values are expressed as mean (standard deviation).

c Values are expressed as median (interquartile range).

d The quantifiable limit was 45 U/L for GGT, 100 U/L for ALP, and 250 U/L for LDH.

### 2.5 Relationships between variables

Clinical experience indicates that the 21 identified variables are not independent. Hence, cluster analysis was performed to confirm the relationships between these variables. In our study, agglomerative clustering algorithms, a hierarchical clustering method, were used to cluster variables. In this method, each variable is initially considered to be its own cluster, and then, the clusters are hierarchically combined, with clusters with the smallest distances being combined first.^13^ This crucial step of hierarchical clustering is required to define the dissimilarity or proximity measure that appropriately quantifies how similar are individuals or variables. Then, a link function was implemented to calculate the distance between two clusters. Here, we chose the correlation between vectors of value function, which is a similarity measure used for clustering variables. The complete‐linkage (or furthest‐neighbor) function, which uses a greatest‐distance metric between clusters, was then selected to perform the cluster analysis. The results were shown in a dendrogram illustrating the relationship between the tested variables.

### 2.6 Identification of DM clusters

Because the dendrogram confirmed the redundancy between the identified variables, PCA was first performed to achieve feature exaction, which can accomplish dimensionality reduction without losing important information about the variables.^13^ Here, we used categorical PCA (CATPCA), which is used for mixed data that include continuous variables and binary variables. CATPCA of the original variables yielded 21 independent components ordered by decreasing eigenvalues or variances. Components with an eigenvalue >1 explained most of the variance, and were retained for further cluster analysis. Based on the PCA‐transformed data, another cluster analysis was conducted to identify DM subgroups. We chose the squared Euclidean distance, which is the most commonly used similarity measure. We implemented the Ward method, which minimizes the total within‐cluster variance. Differences in characteristics between the clusters were assessed using analysis of variance for continuous normally distributed variables, the non‐parametric Kruskal‐Wallis test for non‐normally distributed variables, and the χ^2^ test or Fisher exact test for categorical variables. A *P* value < 0.05 was considered statistically significant.

## 3 RESULTS

### 3.1 Subject characteristics

We enrolled 720 DM patients, of whom 522 (72.5%) were female (Table 1). The mean age at onset was 46.3 ± 14.6 years, and the median duration of disease was 9.0 months (3.0‐33.0 months). The most frequent clinical manifestation was muscle weakness (77.8%), followed by heliotrope rash (71.1%), myalgia/muscle tenderness (56.8%), V‐sign/shawl sign (56.3%), and ILD (53.2%). The co‐occurrence of muscle weakness and ILD occurred in 293 (40.7%) patients. The median CK level was 161.5 U/L (49.0‐746.5 U/L), and an elevated CK level was present in 46.5% of patients. A total of 238 (33.1%) patients received aggressive immunosuppressive therapy with cyclophosphamide, mycophenolate mofetil, cyclosporine, and tacrolimus.

### 3.2 Relationships between variables

**Figure 1:**
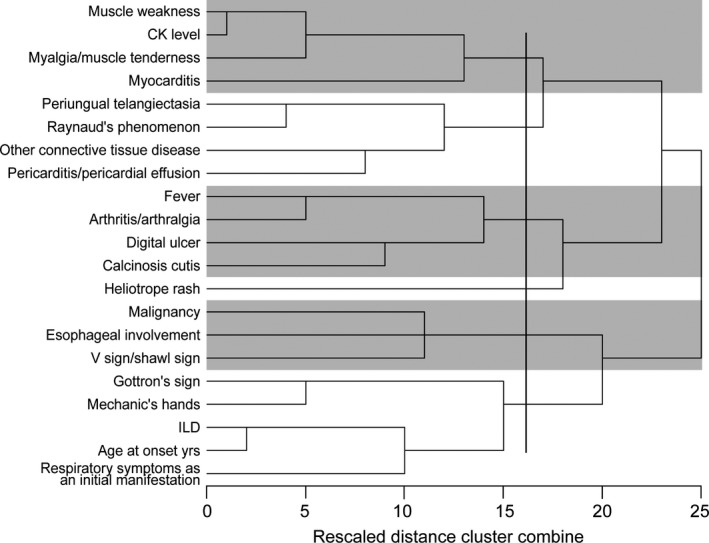
Dendrogram showing the process and results of hierarchical cluster analysis of 21 variables. The horizontal axis represents the rescaled distance cluster combine in which the biggest distance between clusters was marked as 25. The horizontal lines on the left represent the clustering observations, which in our case are clinical variables. The dendrogram shows the process of hierarchical cluster analysis in which variables or clusters join together to form a bigger cluster. Variables or clusters that possess similar distribution patterns join together on the left, while clusters that possess more dissimilar distribution patterns join together on the right. The 21 variables can be optimally divided into 6 groups. ILD, interstitial lung disease; CK, serum creatine kinase

Figure 1 shows the process and results of the hierarchical cluster analysis of the 21 clinical variables. These variables could be optimally divided into six groups, confirming that the variables were not independent, and that the information obtained from these variables was redundant. Hence, these variables could not be directly subjected to cluster analysis.

### 3.3 CATPCA

CATPCA of the original variables yielded 21 independent principal components ordered by decreasing variances. The first 9 components with an eigenvalue >1 explained 54.7% of the variance and were retained for further cluster analysis. The correlations of the 21 variables with these nine components is presented in Table S2, and the last 12 components in Table S3. The most correlated original variables of each component are listed in order in the table. For example, component 1 mostly correlated with ILD, while component two highly correlated with muscle weakness, myalgia/muscle tenderness, and CK level, which can be summarized as muscle involvement.

### 3.4 Cluster analysis of DM patients

**Figure 2:**
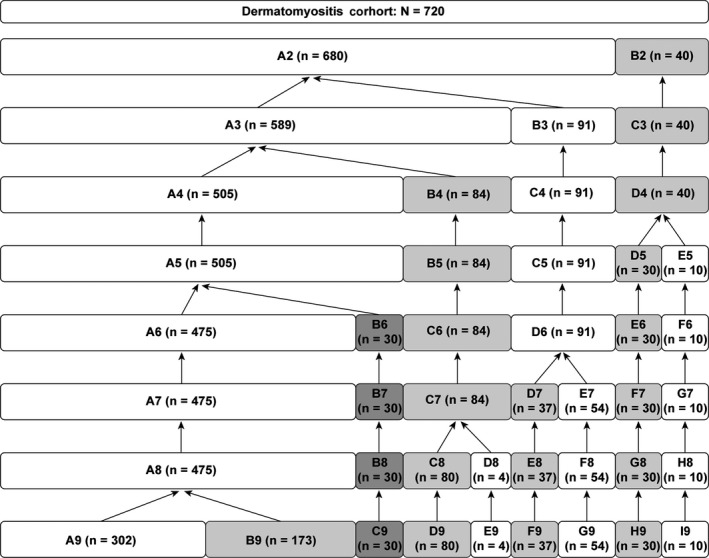
Agglomerative hierarchical clustering of the 720 dermatomyositis patients based on categorical principal components analysis. Agglomerative clustering algorithms start with each individual in its own cluster and then combine clusters hierarchically. Here, we present the process of combination from 9 clusters to 1 cluster. The letters refer to the individual clusters, and the numbers behind the letters refer to the number of clusters in that cycle. The n in the parenthesis indicates the number of patients included in each cluster

Hierarchical cluster analysis was performed among the 720 patients based on the nine principal components derived from the CATPCA. Figure 2 shows the grouping of the patients as the number of clusters decreased from 9 to 1. The clustering that resulted in six groups was chosen for further analysis, in part because of the principle of equipartition, which states that the number of patients in each cluster should be approximately equal. Cluster A5 in the five‐cluster grouping was produced by the combination of the cluster with the largest number of patients (A6) and another cluster (B6) in the six‐cluster grouping rather than by the combination of the two clusters with smaller numbers of patients, which made the six‐cluster grouping the best choice. Furthermore, the clinical characteristics going from six clusters to five, four, or three clusters resulted in patient features that were more homogeneous rather than more distinct. Table 2 shows the clinical characteristics of the six groups. Most of the tested characteristics significantly differed across the six clusters. A summary of characteristics of these six DM clusters is presented in Table 3.

**Table 2:** Clinical characteristics of 720 patients with dermatomyositis according to the 6 clusters identified using principal component analysis‐based cluster analysis

	Cluster A (n = 475)	Cluster B (n = 30)	Cluster C (n = 84)	Cluster D (n = 91)	Cluster E (n = 30)	Cluster F (n = 10)	Overall *P* value
Demographics							
Female,^a^ %	71.4	66.7	70.2	79.1	83.3	70.0	0.437
Age at onset,^b^ years	45.6 (15.2)	56.4 (9.4)	47.3 (13.4)	46.0 (13.3)	47.4 (12.6)	37.9 (12.5)	<0.0001
Clinical features							
Muscle weakness, %	77.5	90.0	71.4	80.2	73.3	100.0	0.148
Myalgia/muscle tenderness, %	57.5	46.7	45.2	68.1	50.0	70.0	0.033
Heliotrope rash, %	73.7	70.0	64.3	71.4	53.3	60.0	0.121
Gottron sign, %	41.9	33.3	57.1	60.4	43.3	40.0	0.003
V‐sign/shawl sign, %	60.6	70.0	42.9	48.4	43.3	30.0	0.001
Mechanic's hand, %	0.2	10.0	2.4	70.3	6.7	0.0	<0.0001
Raynaud phenomenon, %	8.2	6.7	14.3	19.8	10.0	40.0	0.003
Periungual telangiectasia, %	0.0	6.7	0.0	37.4	0.0	0.0	<0.0001
Digital ulcer, %	0.2	0.0	21.4	7.7	0.0	0.0	<0.0001
Calcinosis cutis, %	0.0	0.0	4.8	0.0	0.0	0.0	0.002
Fever, %	25.7	23.3	31.0	22.0	43.3	30.0	0.256
Arthritis/arthralgia, %	34.9	26.7	28.6	34.1	46.7	30.0	0.524
Interstitial lung disease, %	46.9	50.0	72.6	60.4	70.0	80.0	<0.0001
Respiratory symptoms as an initial manifestation, %	1.7	3.3	73.8	11.0	0.0	10.0	<0.0001
Pericarditis/pericardial effusion, %	2.5	13.3	13.1	12.1	16.7	20.0	<0.0001
Myocarditis, %	0.0	0.0	0.0	0.0	0.0	100.0	<0.0001
Esophageal involvement, %	17.1	30.0	23.8	19.8	6.7	10.0	0.138
Comorbidities							
Malignancy, %	0.0	100.0	0.0	0.0	0.0	10.0	<0.0001
Other connective tissue disease, %	0.0	0.0	2.4	6.6	100.0	10.0	<0.0001
Laboratory data							
Creatine kinase level,^c^ U/L	180.0 (726.0)	114.0 (1204.3)	81.0 (395.2)	174.0 (1173.6)	96.59 (735.3)	92.0 (5735.4)	0.082
Use of aggressive immunosuppressive therapy,^a^ %	28.0	33.3	46.4	38.5	63.3	20.0	<0.0001

a Variables not used for the creation of clusters.

b Values are expressed as mean (standard deviation).

c Values are expressed as median (interquartile range).

**Table 3:** Description of the 6 dermatomyositis clusters identified using categorical principal component analysis‐based cluster analysis

	Cluster A (n = 475)	Cluster B (n = 30)	Cluster C (n = 84)	Cluster D (n = 91)	Cluster E (n = 30)	Cluster F (n = 10)
Age	Middle‐aged^a^	Old	Middle‐aged	Middle‐aged	Middle‐aged	Young
Muscular involvement^b^	Frequent	Very frequent	Moderate	Very frequent	Moderate	All
Interstitial lung disease	Moderate	Moderate	Frequent	Frequent	Frequent	Very frequent
Respiratory symptoms as an initial manifestation	Rare	Rare	Very frequent	Moderate	Rare	Moderate
Prominent skin lesions	None	V‐sign/shawl sign	Digital ulcer Gottron sign Calcinosis cutis	Mechanic's hands Gottron sign Raynaud phenomenon Periungual telangiectasia	None	Raynaud phenomenon
Other comorbidities	None	Malignancy	None	None	Other CTD	Myocarditis
Use of aggressive immunosuppressive therapy	Moderate	Moderate	Frequent	Frequent	Very frequent	Moderate

Abbreviation: CTD, connective tissue disease.

a The average age ranged from 45.6 to 47.4 years.

b Taking into account muscle weakness, myalgia/muscle tenderness and creatine kinase level.

Cluster A (n = 475) was the largest group in the present DM cohort. Patients in this cluster showed intermediate frequencies for common manifestations and almost no rare manifestations. Figure 2 shows that cluster A was composed of two sub‐clusters (n_A9 = _302, n_B9_ = 173) in the process of going from nine clusters to eight clusters. The details of these two sub‐clusters are presented in Table S4. Significant differences were found between these two subgroups. Sub‐cluster A9 included patients with the 2nd highest rate of muscle weakness (83.1%), the highest rate of myalgia/muscle tenderness (68.9%), and similar frequencies of heliotrope rash, Gottron sign, and V‐sign/shawl sign (59.6%, 53.3%, and 67.2%). Sub‐cluster B9 contained younger patients (mean age at onset, 41.5 years) with less frequent fever, muscle involvement, arthritis/arthralgia, and ILD, more frequent esophageal involvement, and the highest rate of heliotrope rash (98.3%). Based on these characteristics, we labeled cluster A as “classical DM and classical DM with minimal organ involvement”.

Cluster B (n = 30) included almost all patients with malignancy in the present cohort. They were, on average, the oldest patients (mean age at onset, 56.4 years) with the lowest rate of females, the 2nd highest rate of muscle involvement, moderate ILD, frequent esophageal involvement, frequent V‐sign/shawl sign, and the lowest rate of Gottron sign. Accordingly, we labeled this cluster as “DM with malignancy comorbidities”.

Cluster C (n = 84) was characterized by respiratory involvement, irreversible skin damage, and the lowest rate of muscle weakness (71.4%) and myalgia (45.2%). We found that 73.8% of patients in this cluster showed respiratory symptoms as an initial manifestation, and 72.6% patients had ILD. Patients in this cluster showed the highest rate of digital ulcer (21.4%), and the 2nd highest rate of Gottron sign (57.1%). Cluster C included all cases of calcinosis cutis in our cohort. Accordingly, we labeled this cluster “CADM with ILD”.

Cluster D (n = 91) included patients who had prominent lung, muscle, and skin involvement. Patients in this cluster frequently had ILD (60.4%) and Raynaud phenomenon (19.8%) and very frequently had muscle weakness (80.2%) and myalgia (68.1%). Additionally, these patients had the highest rates of mechanic's hand (70.3%), Gottron sign (60.4%), and periungual telangiectasia (37.4%). Accordingly, we labeled this cluster “DM with dominant lung, muscle, and skin involvement”.

Cluster E (n = 30) included patients with DM accompanied by other connective tissue diseases, which is also known as overlapping connective tissue disease syndromes. These patients showed the highest rate of females (85.3%), fever (43.3%), and arthritis/arthralgia (46.7%), moderate muscular weakness (73.3%), and frequent ILD (70.0%). Accordingly, we labeled this cluster “Overlapping syndromes”.

Cluster F (n = 10) included DM patients with severe cardiac involvement. All these patients had myocarditis and 20% of them had pericarditis/pericardial effusion. They were, on average, the youngest patients (mean age at onset, 37.9 years) with 100% muscular weakness, the highest rate of ILD (80.0%), the highest rate of Raynaud phenomenon (40.0%), and the lowest rate of V‐sign/shawl sign (30.0%). Hence, we labeled this cluster as “DM with dominant cardiomyopathy”.

### 3.5 Relationship between immunosuppressive therapy and clusters

To validate the classification, we examined the relationship between the clusters and immunosuppressive therapy, which is a parameter that was not used for the creation of the clusters and reflects the physicians' clinical judgements of the outcomes. The results are presented in Table 2. As expected, there were significant differences in immunosuppressive therapy across the six clusters (*P* < 0.0001). The ranking from the highest to the lowest rate of aggressive immunosuppressive therapy was as follows: clusters E, C, D, B, A, and F (63.3%, 46.4%, 38.5%, 33.3%, 28.0%, and 20.0%, respectively).

## 4 DISCUSSION

In this study, we applied PCA‐based cluster analysis to analyze the clinical data of a large group of DM patients, which eventually resulted in the identification of 6 subgroups: cluster A, classical DM and classical DM with minimal organ involvement; cluster B, older DM patients with malignancies; cluster C, amyopathic/hypomyopathic DM patients with ILD and skin ulcers; cluster D, DM patients with prominent lung, muscle, and skin involvement; cluster E, DM patients with other connective tissue diseases; and cluster F, DM patients with severe cardiomyopathy. Our results indicated that a variety of clinical manifestations are valuable in the subtyping of DM, which emphasizes the need for the multidimensional assessment of DM patients.

We found that some of the six distinct subgroups identified in our study were highly consistent with classes or specific subtypes of DM defined by previous classification criteria and studies. The characteristics of cluster B (DM with malignancy) and cluster E (overlapping syndromes) meet the 1975 classification criteria for DM associated with cancer and DM associated with other connective tissue diseases proposed by Bohan and Peter.^3^, ^4^ Cluster E showed the highest rate of aggressive immunosuppressive therapy (63.3%), which may be attributed to the extra immunosuppressive treatment required for the other connective tissue diseases present in this cluster. Cluster C (CADM with ILD) was characterized by prominent ILD, skin ulcers, and Gottron sign with minimal muscle involvement. Generally, these features fit well with those of previously reported CADM with positive anti‐MDA5 antibody.^14^, ^15^ The prognosis of anti‐MDA5‐positive CADM patients is unfavorable, with a 40% mortality rate, attributed mostly to the rapid progression of ILD.^15^ In our study, we found that the patients in cluster C were more likely to receive aggressive immunosuppressive therapy (46.4%), which reflected the physicians’ clinical judgements of a poorer outcome in this cluster. Cluster D (DM with dominant lung, muscle, and skin involvement) was characterized by myositis, ILD, mechanic's hand, and Raynaud phenomenon, which was consistent with the clinical manifestations of antisynthetase syndrome. The first case series of patients with antisynthetase syndrome was published in 1990, which defined the disease as a constellation of the following signs: polymyositis, interstitial pneumonia, Raynaud phenomenon, mechanic's hand, and arthritis.^16^ However, cluster D in our study had the highest rate of periungual telangiectasia and Gottron sign among all the clusters, and these manifestations have scarcely been reported in patients with antisynthetase syndrome.

In addition to the clusters consistent with known DM subtypes, our analysis identified a subgroup characterized by cardiomyopathy (cluster F). At present, cardiac involvement is regarded as a complication of DM, and DM with cardiac involvement has never been reported as a distinct subtype. However, our study revealed that this subgroup had distinguishable features separating it from the overall DM population. For example, most patients in this cluster exhibited muscle weakness, myalgia/muscle tenderness, and elevated serum CK levels. One study of 16 patients with biopsy‐proven myositis also found that all patients with active myocarditis had skeletal muscle involvement.^17^ Furthermore, a high prevalence of Raynaud phenomenon and ILD were observed in this subgroup. In a systematic review on cardiac involvement in adult IIM patients, Zhang et al found there was no correlation between overall disease severity and cardiac involvement.^18^ Given the phenotypic uniqueness of this subgroup and the discordance between cardiac involvement and disease severity, we propose that DM with myocarditis be regarded as a new distinct subtype of DM. Anti‐Ro antibody is reported to be a biomarker specifically associated with cardiac involvement in DM,^19^ which provides mechanistic evidence in favor of our findings.

We also performed cluster analysis of variables, resulting in a dendrogram. Variables categorized into the same groups were more closely associated with each other than with other variables, and had similar distribution patterns among patients. Our results were mostly consistent with those of previous studies. Muscle involvement and myocarditis shared similar patterns of distribution; the association of these two conditions was also observed in cluster F, as has been discussed above. ILD was associated with Gottron sign and mechanic's hand. This association was also demonstrated in cluster D, and is consistent with the results of previous studies.^20^ The presence of comorbidities such as other connective tissue diseases was associated with Raynaud phenomenon, periungual telangiectasia, and pericardial effusion, which is consistent with a previous review stating that Raynaud phenomenon is one of the most frequently reported symptoms in mixed connective tissue disease (MCTD), while serositis and vasculitis are occasionally encountered in MCTD.^21^

PCA showed that ILD and muscle involvement were the variables with the highest component loading (highest eigenvalue) in the 1st and 2nd principal components, respectively. These two factors served as the primary driving force in the clustering of our cohort of DM patients, while other clinical variables played a secondary role. Thus, we should pay attention to the screening and assessment of ILD in clinical practice.

This study represents the first‐ever attempt to apply cluster analysis to a cohort of DM patients. Due to the intrinsic property of this method, a large sample size is required. Hence, the method has been applied to patient populations of certain common diseases, such as chronic obstructive pulmonary disease, heart failure, encephalitis, and Parkinson disease.^22^, ^23^, ^24^, ^25^, ^26^ However, it has scarcely been used in the field of rheumatology.^21^, ^27^, ^28^ Only one study has used cluster analysis to group 233 patients with antisynthetase syndrome. That study resulted in three clusters and revealed that the tropism of the disease depends more on muscle involvement in the case of patients with anti‐Jo‐1 antibodies and more on ILD in the case of patients with anti‐PL7 or anti‐PL12 antibodies. Consequently, the mortality (due to ILD) is higher in the anti‐PL7/12 group than in anti‐Jo‐1 group.^27^

We conducted PCA to transform the original variables included in the cluster analysis for two reasons. First, PCA is especially useful to reduce dimensionality, which can eliminate noisy variables that may corrupt the cluster structure.^13^ Independence of the variables is a prerequisite for cluster analysis, and clinically, we were aware that the original variables lacked independence. Accordingly, in a preliminary analysis, the direct application of cluster analysis to the original variables did not yield satisfactory results. Second, PCA not only reduced dimensionality but also detected key features of the data.^13^ Studies utilizing cluster analysis have explored various methods of pre‐processing the original variables, including factor analysis,^22^ PCA,^23^ and the subjective deletion of variables with a prevalence of <20% or >80%.^24^ PCA stands out from all these pre‐processing methods, as it maintains the integrity of the data, and consequently, would not leave out information on symptoms with a lower prevalence. DM is characterized by its heterogeneity of symptoms. Some symptoms are less prevalent but are nevertheless clinically significant; if these symptoms had been missed due to methodological flaws, the reliability of clustering would have been compromised. Furthermore, our results proved correct our notion that PCA‐based cluster analysis would be suitable for the subtyping of DM, a disease with several rare but important symptoms.

Our study has several strengths. The large study sample of over 700 DM patients made it possible to demonstrate diverse phenotypes and to conduct the 1st cluster analysis in the field of DM. The clustering resulted in 6 subgroups, most of which showed good concordance with previous reports. Furthermore, new subgroups and features emerged, providing a basis for further studies. There are also several limitations of our study. First, missing data and memory bias existed due to the retrospective nature of the study. For example, cardiomyopathy was only detected when patients were referred for echocardiography due to relevant clinical manifestations or abnormal electrocardiographic findings, which precluded the detection of subclinical cardiac involvement. Second, our study did not include myositis‐specific antibody profiles because the detecting kits were not commercially available until October 2015. Hence, most of the patients lacked these data. We believe that myositis‐specific antibodies will greatly facilitate DM subtyping in future studies. Third, the diversity of six DM subgroups obtained using cluster analysis needs to be validated by long‐term follow‐up studies, and the universality of the classification also needs to be validated in an independent cohort. However, our results did identify some important prognostic factors that have been reported in previous studies, and we analyzed the clinicians’ therapeutic choices as a surrogate end‐point measure, which provided some support for the validity of the subgrouping.

In conclusion, we, for the first time, applied a new exploratory statistical methodology to a large cohort of DM patients, which led to the identification of six clinical subgroups of DM. These subgroups may help to develop individualized treatments and improve patient prognosis. Longitudinal studies are needed to evaluate the prognostic value of the classification.

## CONFLICT OF INTEREST

The authors declare they have no potential conflicts of interest with respect to the research, authorship, and/or publication of this article.

## AUTHOR CONTRIBUTION

Huiyi Zhu, Qian Wang and Nan Jiang were the authors who contributed to interpretation of data, completed drafting of the article and critically revised it. Chanyuan Wu was in charge of original data collection for forming the entire cohort. Yanhong Wang, Jiuliang Zhao, and Dong Xu contributed to the basic conception and design of the study. Qian Wang, Mengtao Li, and Xiaofeng Zeng contributed greatly to the reading, revision and approval of the final version.
