# Supplementary material for: Identification of 6 dermatomyositis subgroups using principal component analysis‐based cluster analysis
Source: Int J Rheum Dis. 2019 Jun 9;22(8):1383–92. doi: 10.1111/1756-185X.13609 (PMC6771972; doi:10.1111/1756-185X.13609)
Supplement: Supplementary file 1 [file APL-22-1383-s001.docx]

**Supplementary Material**

**Supplementary** **Table S1.** Comparison of characteristics of dermatomyositis patients included in and excluded from the PCA-based cluster analysis

|  | **Included Patients, N (%)**  **(n = 720)** | **Excluded Patients, N (%)**  **(n = 74)** | **P-value** |
| --- | --- | --- | --- |
| **Demographics** |  |  |  |
| Female | 522 (72.5) | 57 (77.1) | 0.4 |
| Age at onset^§^, years | 46.3 (14.6) | 45.48 (12.4) ^‡^ | 0.68 |
| Course of disease^¶^, months | 9.0 (30.0) ^†^ | 12.0 (45.0) ^‡^ | 0.05 |
| **Clinical features** |  |  |  |
| Muscle weakness | 560 (77.8) | 45 (67.1) ^‡^ | 0.05 |
| Myalgia/muscle tenderness | 409 (56.8) | 30 (44.7) ^‡^ | 0.06 |
| Heliotrope rash | 512 (71.1) | 50 (67.5) | 0.52 |
| Gottron sign | 329 (45.7) | 38 (51.4) | 0.35 |
| V sign/shawl sign | 405 (56.3) | 44 (59.5) | 0.6 |
| Mechanic’s hand | 72 (10.0) | 15 (20.3) | 0.007 |
| Raynaud phenomenon | 78 (10.8) | 13 (17.5) | 0.08 |
| Periungual telangiectasia | 36 (5.0) | 6 (8.1) | 0.26 |
| Digital ulcer | 26 (3.6) | 4 (5.4) | 0.44 |
| Calcinosis cutis | 4 (0.6) | 2 (2.7) | 0.04 |
| Fever | 191 (26.5) | 18 (34.0) ^‡^ | 0.24 |
| Arthritis/arthralgia | 246 (34.2) | 21 (31.3) ^‡^ | 0.64 |
| Interstitial lung disease | 383 (53.2) | 44 (63.7) ^‡^ | 0.09 |
| Respiratory symptoms as an initial manifestation | 82 (11.4) | 14 (19) | 0.06 |
| Pericarditis/pericardial effusion | 45 (6.3) | 5 (8.6) ^‡^ | 0.48 |
| Myocarditis | 10 (1.4) | 1 (1.7) ^‡^ | 0.84 |
| Esophageal involvement | 131 (18.2) | 15 (25.4) ^‡^ | 0.17 |
| **Comorbidities** |  |  |  |
| Malignancy | 31 (4.3) | 3 (4.1) | 0.91 |
| Other connective tissue disease | 39 (5.4) | 7 (9.4) | 0.16 |
| **Laboratory data** |  |  |  |
| Creatine kinase level^¶^, U/L | 161.5 (697.5) | 133.0 (787.0) ^‡^ | 0.89 |
| Elevated GGT or ALP | 249 (38.7) ^†^ | 27 (41.5) ^‡^ | 0.67 |
| Elevated LDH | 537 (77.3) ^†^ | 42 (67.7) ^‡^ | 0.09 |

†Based on available data (n = 694, 643, and 659, respectively)

‡Based on available data (n = 60, 67, 67, 67, 53, 67, 69, 58, 58, 59, 44, 65, and 62, respectively)

§Values are expressed as mean (standard deviation).

¶Values are expressed as median (interquartile range).

**Supplementary** **Table S2.** Correlations of the 21 variables with the nine principal components derived from the categorical principal component analysis in the 720 DM subjects.

|  | **Components** | | | | | | | | |
| --- | --- | --- | --- | --- | --- | --- | --- | --- | --- |
|  | **1** | **2** | **3** | **4** | **5** | **6** | **7** | **8** | **9** |
| **Interstitial lung disease** | **0.474** | -0.31 | 0.363 | -0.146 | 0.233 | -0.015 | -0.14 | 0.009 | 0.037 |
| **Creatine kinase level** | -0.023 | **0.549** | 0.268 | -0.149 | -0.077 | 0.155 | -0.134 | -0.021 | -0.129 |
| **Muscle weakness** | -0.187 | **0.507** | 0.308 | 0.002 | 0.256 | 0.163 | -0.171 | -0.085 | -0.032 |
| **Myalgia/muscle tenderness** | -0.013 | **0.496** | -0.039 | 0.139 | 0.391 | 0.208 | -0.139 | 0.042 | -0.169 |
| **Age at onset (years)** | -0.092 | -0.329 | 0.517 | 0.116 | 0.328 | -0.092 | 0.123 | 0.123 | -0.007 |
| **Arthritis/arthralgia** | 0.381 | -0.052 | -0.472 | -0.056 | 0.396 | 0.04 | 0.123 | 0.134 | 0.009 |
| **Periungual telangiectasia** | 0.225 | 0.234 | 0.094 | 0.478 | -0.198 | -0.086 | 0.407 | 0.204 | 0.016 |
| **Digital ulcer** | 0.268 | 0.045 | -0.171 | 0.468 | -0.116 | -0.344 | -0.153 | -0.072 | -0.019 |
| **Fever** | 0.327 | 0.003 | -0.025 | -0.181 | 0.504 | -0.265 | -0.1 | -0.08 | -0.317 |
| **Gottron sign** | 0.395 | -0.202 | 0.105 | 0.303 | 0.066 | 0.475 | -0.063 | -0.213 | 0.101 |
| **Respiratory symptoms as an initial manifestation** | 0.202 | -0.27 | 0.232 | 0.12 | -0.152 | -0.217 | -0.592 | 0.001 | 0.072 |
| **Heliotrope rash** | -0.235 | 0.013 | -0.272 | -0.186 | 0.08 | -0.122 | -0.222 | 0.661 | 0.081 |
| **Mechanic's hands** | 0.331 | 0.059 | 0.222 | 0.161 | -0.033 | 0.438 | -0.026 | 0.453 | 0.067 |
| **Myocarditis** | 0.15 | 0.237 | 0.065 | -0.377 | -0.018 | -0.114 | -0.05 | -0.363 | 0.507 |
| **Malignancy** | -0.208 | -0.011 | 0.298 | 0.019 | 0.296 | -0.22 | 0.344 | 0.158 | 0.501 |
| **Other connective tissue disease** | 0.286 | 0.083 | 0.073 | -0.172 | -0.13 | -0.232 | 0.393 | -0.066 | -0.465 |
| **V sign/shawl sign** | -0.312 | -0.133 | -0.093 | 0.301 | 0.342 | 0.087 | 0.23 | -0.315 | 0.025 |
| **Pericarditis/pericardial effusion** | 0.342 | 0.237 | 0.325 | -0.177 | -0.018 | -0.238 | 0.176 | 0.145 | 0.023 |
| **Calcinosis cutis** | 0.088 | 0.134 | -0.382 | 0.212 | 0.288 | -0.175 | -0.14 | 0.005 | 0.148 |
| **Esophageal involvement** | -0.253 | 0.208 | 0.279 | 0.396 | 0.039 | -0.393 | -0.217 | -0.017 | -0.103 |
| **Raynaud phenomenon** | 0.399 | 0.398 | -0.2 | 0.079 | -0.089 | -0.093 | 0.03 | -0.045 | 0.344 |

The first nine components, with eigenvalues >1, were selected for the further cluster analysis. The variance of the nine components were 7.6, 7.4, 7.1, 5.8, 5.7, 5.5, 5.5, 5.1 and 5.0%. Complementary data of the last 12 components are shown in the supplementary material.

**Supplementary Table S3.** Correlations of the 21 variables with the last 12 principal components derived from the categorical principal component analysis in the 720 DM subjects.

|  | **Components** | | | | | | | | | | | |
| --- | --- | --- | --- | --- | --- | --- | --- | --- | --- | --- | --- | --- |
|  | **10** | **11** | **12** | **13** | **14** | **15** | **16** | **17** | **18** | **19** | **20** | **21** |
| Interstitial lung disease | 0.106 | 0.175 | 0.087 | -0.117 | 0.325 | -0.132 | 0.073 | -0.113 | -0.152 | -0.196 | 0.124 | 0.411 |
| Creatine kinase level | -0.092 | -0.214 | -0.037 | -0.094 | 0.517 | -0.033 | 0.101 | -0.314 | 0.022 | 0.005 | -0.248 | -0.186 |
| Muscle weakness | -0.188 | 0.27 | 0.054 | -0.131 | -0.035 | 0.193 | 0.126 | 0.347 | -0.154 | -0.198 | 0.312 | -0.144 |
| Myalgia/muscle tenderness | 0.161 | 0.122 | -0.203 | 0.496 | -0.147 | -0.042 | -0.055 | -0.068 | 0.175 | 0.003 | -0.123 | 0.287 |
| Age at onset | -0.093 | 0.205 | -0.022 | 0.27 | 0.202 | 0.055 | -0.174 | 0.023 | -0.124 | 0.45 | -0.011 | -0.199 |
| Arthritis/arthralgia | -0.046 | 0.017 | -0.276 | -0.073 | 0.207 | -0.12 | 0.086 | -0.055 | 0.332 | 0.026 | 0.34 | -0.238 |
| Periungual telangiectasia | 0.286 | 0.215 | -0.084 | 0.003 | 0.067 | -0.053 | -0.299 | -0.104 | -0.156 | -0.327 | 0.013 | -0.169 |
| Digital ulcer | -0.394 | 0.062 | -0.349 | 0.07 | -0.001 | -0.055 | 0.367 | -0.009 | -0.283 | 0.061 | -0.087 | 0.051 |
| Fever | 0.143 | -0.185 | -0.108 | -0.278 | -0.29 | 0.203 | -0.169 | -0.069 | -0.226 | -0.034 | -0.216 | -0.133 |
| Gottron sign | -0.272 | 0.103 | 0.021 | -0.201 | -0.034 | -0.107 | -0.178 | 0.238 | 0.237 | -0.05 | -0.351 | -0.048 |
| Respiratory symptoms as an initial manifestation | 0.298 | -0.022 | 0.033 | 0.266 | 0.004 | 0.201 | 0.141 | 0.039 | 0.258 | -0.19 | -0.011 | -0.259 |
| Heliotrope rash | 0.069 | 0.316 | 0.017 | -0.155 | 0.088 | -0.174 | 0.07 | 0.22 | -0.04 | -0.027 | -0.314 | -0.027 |
| Mechanic’s hands | 0.084 | -0.118 | 0.186 | -0.096 | -0.37 | -0.022 | 0.304 | -0.225 | -0.104 | 0.198 | 0.093 | -0.066 |
| Myocarditis | 0.091 | 0.344 | -0.048 | 0.042 | -0.236 | -0.332 | 0.008 | -0.178 | -0.064 | 0.088 | -0.049 | -0.165 |
| Malignancy | -0.211 | -0.119 | -0.095 | -0.049 | -0.095 | 0.284 | 0.163 | -0.144 | 0.231 | -0.218 | -0.146 | 0.086 |
| Other connective tissue disease | -0.071 | 0.382 | 0.315 | 0.045 | -0.069 | 0.091 | 0.286 | 0.001 | 0.259 | 0.028 | -0.123 | -0.014 |
| V sign/shawl sign | 0.452 | -0.107 | 0.131 | -0.04 | 0.124 | -0.193 | 0.411 | 0.132 | -0.097 | -0.012 | -0.132 | -0.082 |
| Pericarditis/pericardial effusion | -0.042 | -0.466 | 0.025 | 0.198 | -0.036 | -0.367 | -0.002 | 0.429 | 0.015 | -0.028 | 0.013 | -0.007 |
| Calcinosis cutis | -0.269 | -0.088 | 0.669 | 0.192 | 0.064 | -0.056 | -0.141 | -0.147 | -0.08 | -0.08 | 0.013 | -0.056 |
| Esophageal involvement | 0.062 | 0.009 | 0.059 | -0.412 | -0.094 | -0.269 | -0.109 | -0.085 | 0.318 | 0.19 | 0.133 | 0.11 |
| Raynaud phenomenon | 0.28 | -0.002 | 0.075 | -0.127 | 0.204 | 0.405 | -0.03 | 0.221 | 0.019 | 0.344 | -0.03 | 0.153 |

The last 13 components, with eigenvalues <1, were excluded from further cluster analysis.

**Supplementary** **Table S4.** Clinical characteristics of the patients with dermatomyositis in cluster A9 and cluster B9, which join together to form cluster A in the cluster analysis.

|  | **Cluster A9**  **(n = 302)** | **Cluster B9**  **(n = 173)** | **p-value** |
| --- | --- | --- | --- |
| **Demographics** |  |  |  |
| Female**^†^**, % | 72.8 | 68.8 | 0.346 |
| Age at onset^‡^, years | 46.5 (9.4) | 41.5 (14.7) | 0.010 |
| **Clinical features** |  |  |  |
| Muscle weakness, % | 83.1 | 67.6 | <0.0001 |
| Myalgia/muscle tenderness, % | 68.9 | 37.6 | <0.0001 |
| Heliotrope rash, % | 59.6 | 98.3 | <0.0001 |
| Gottron sign, % | 53.3 | 22.0 | <0.0001 |
| V sign/shawl sign, % | 67.2 | 49.1 | <0.0001 |
| Mechanic's hands, % | 0.3 | 0.0 | 1.000 |
| Raynaud phenomenon, % | 12.3 | 1.2 | <0.0001 |
| Periungual telangiectasia, % | 0.0 | 0.0 | 1.000 |
| Digital ulcer, % | 0.3 | 0.0 | 1.000 |
| Calcinosis cutis, % | 0.0 | 0.0 | 1.000 |
| Fever, % | 36.4 | 6.9 | <0.0001 |
| Arthritis/arthralgia, % | 40.4 | 25.4 | 0.001 |
| Interstitial lung disease, % | 55.6 | 31.8 | <0.0001 |
| Respiratory symptoms as an initial manifestation, % | 1.3 | 2.3 | 0.470 |
| Pericarditis/pericardial effusion, % | 4.0 | 0.0 | 0.011 |
| Myocarditis, % | 0.0 | 0.0 | 1.000 |
| Esophageal involvement, % | 12.6 | 24.9 | 0.001 |
| **Comorbidities** |  |  |  |
| Malignancy, % | 0.0 | 0.0 | 1.000 |
| Other connective tissue disease, % | 0.0 | 0.0 | 1.000 |
| **Laboratory data** |  |  |  |
| Creatine kinase level^§^, U/L | 177.5 (678.8) | 192.0 (1054.5) | 0.677 |
| Use of aggressive immunosuppressive therapy**^†^**, % | 29.8 | 24.9 | 0.248 |

**†**Variables not used for the creation of the clusters.

‡Values are expressed as mean (standard deviation).

§Values are expressed as median (interquartile range).
